# Semax, a Synthetic Regulatory Peptide, Affects Copper-Induced Abeta Aggregation and Amyloid Formation in Artificial Membrane Models

**DOI:** 10.1021/acschemneuro.1c00707

**Published:** 2022-01-26

**Authors:** Michele F.M. Sciacca, Irina Naletova, Maria Laura Giuffrida, Francesco Attanasio

**Affiliations:** Consiglio Nazionale delle Ricerche, Istituto di Cristallografia, Via Paolo Gaifami, 18, Catania 95126, Italy

**Keywords:** amyloid β, copper, Alzheimer, peptides, aggregation, oligomers

## Abstract

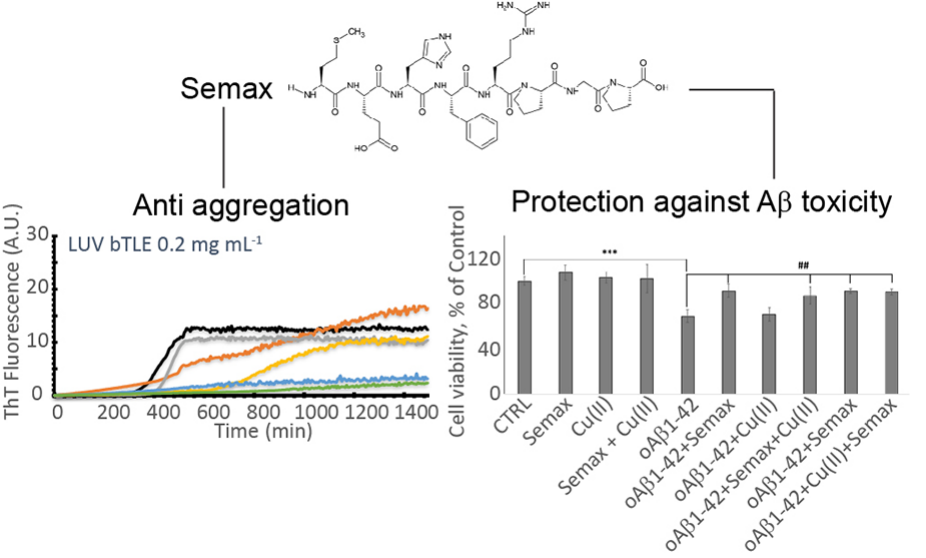

Alzheimer’s disease, the most common form of dementia, is characterized by the aggregation of amyloid beta protein (Aβ). The aggregation and toxicity of Aβ are strongly modulated by metal ions and phospholipidic membranes. In particular, Cu^2+^ ions play a pivotal role in modulating Aβ aggregation. Although in the last decades several natural or synthetic compounds were evaluated as candidate drugs, to date, no treatments are available for the pathology. Multifunctional compounds able to both inhibit fibrillogenesis, and in particular the formation of oligomeric species, and prevent the formation of the Aβ:Cu^2+^ complex are of particular interest. Here we tested the anti-aggregating properties of a heptapeptide, Semax, an ACTH-like peptide, which is known to form a stable complex with Cu^2+^ ions and has been proven to have neuroprotective and nootropic effects. We demonstrated through a combination of spectrofluorometric, calorimetric, and MTT assays that Semax not only is able to prevent the formation of Aβ:Cu^2+^ complexes but also has anti-aggregating and protective properties especially in the presence of Cu^2+^. The results suggest that Semax inhibits fiber formation by interfering with the fibrillogenesis of Aβ:Cu^2+^ complexes.

## Introduction

Alzheimer’s disease represents the most common neurodegenerative disease.^1^ According to data from the World Alzheimer Report, over 46.8 million people were affected by dementia worldwide in 2015, with a prevision of a doubling of this number in the next 20 years.^2,3^ To date, despite the intense research activity, the etiology of the pathology is not fully understood.

The principal hallmark of Alzheimer’s disease is the appearance in the hippocampal region of the brain^4^ of proteinaceous deposits inside neuronal cells, called neurofibrillary tangles, mainly constituted by fibrillar aggregates of phosphorylated tau protein,^5^ and in the extracellular space, called amyloid plaques, mainly constituted by fibrillar aggregates of amyloid beta protein (Aβ).^6^ Amyloid β protein is the final product of the aberrant cleavage of amyloid precursor protein (APP).^7^

It was proposed that an abnormally high concentration of Aβ could result in aggregation into a β-sheet rich structure, the starting point of the fibrillogenesis of Aβ.^8^ Aggregation is a complex mechanism that starts with the formation of oligomeric species, suggested to be the more toxic species for cells,^9,10^ that undergo conformational reorganization into protofibrils and fibrils. Monomeric and fibrillar forms have been demonstrated to be, respectively, protective^11^ or mostly inert^12^ for neuronal cells. It is widely recognized that metal ions, in particular copper, are involved in the aggregation process of several protein and amyloidogenic peptides.^13−15^

A pivotal role in Aβ fibrillogenesis is played by phospholipidic membranes and metal ions.^16−19^ Neuronal cell membranes are not only the target of amyloid toxicity but also an active actor that can drive Aβ toward the formation of toxic species.^20−24^ Great efforts were put in evaluating the role of metal ions, in particular Cu^2+^ and to a lesser extent Zn^2+^, considering the importance of these two metal ions in the normal brain function and the presence of these metals in the amyloid plaques.^25−27^ Over the years, a great amount of data has been collected on the interaction and complex formation of Aβ and Cu^2+^. Although results are often contradictory, there is a general consensus that the Aβ/Cu^2+^ complex formation drives and modulates the final end of the aggregation process.^16−19^ It was demonstrated that, depending on the protein/ion ratio, the aggregation is driven toward amorphous or fibrillar structures.^17,28−32^ In the last decade, many peptide inhibitors of Aβ aggregation have been proposed^33−36^ and evaluated for the treatment of AD.^37^ Peptides capable of both preventing fibril formation and sequestering free Cu^2+^ ion are particularly interesting.^38−45^

Heptapeptide ACTH(4–7)-PGP, also known as Semax (Met-Glu-His-Phe-Pro-Gly-Pro), is an ACTH-like peptide, in particular an analog of the endogenous regulatory peptide ACTH (4–10) (Met-Glu-His-Phe-Arg-Trp-Gly); biological effect studies on Semax showed that this peptide has pronounced nootropic, neuroprotective, and neurotrophic properties,^46−50^ stimulating learning and memory formation in rodents and humans.^46,51,52^ It has been suggested that these effects of Semax are associated with its ability to modulate the expression of neurotrophins.^53,54^ Semax’s ability to form stable complexes with copper(II) and to prevent the copper-induced cytotoxicity on SH-SY5Y neuroblastoma and RBE4 endothelial cell lines was also investigated.^55,56^ The marked nootropic, neuroprotective, and neurotrophic effects of Semax could make this peptide a promising candidate for the prevention and treatment of pathologies of the central nervous system.

To date, there is a lack of information about the ability of this peptide to inhibit or hamper Aβ fibril formation. Here, we performed a combination of biophysical and biological experiments to test the ability of Semax to modulate fiber formation in both the presence and absence of Cu^2+^ and model membranes. We found that Semax is able, in a concentration-dependent way, to inhibit fiber formation both in the buffer and in the presence of model membranes. Differential scanning calorimetry allowed us to evaluate how Semax modulates over time the interaction of amyloid β-1-40 (Aβ_1–40_) with the hydrophobic core of phospholipidic membranes in the presence of Cu^2+^ ions. Finally, we show that Semax exerts its anti-aggregation properties and is able to prevent membrane disruption, especially in the presence of metal ions. The results suggest that Semax could modulate the Aβ fiber formation process. Although more in-depth details are needed, this first study about the anti-aggregating properties of Semax provides promising results and could represent the starting point for a complete evaluation of Semax as an anti-AD drug candidate.

## Results and Discussion

### Thioflavin T Assay in the Buffer Reveals Anti-aggregating Properties of Semax in the Presence of Cu^2+^

**Figure 1:**
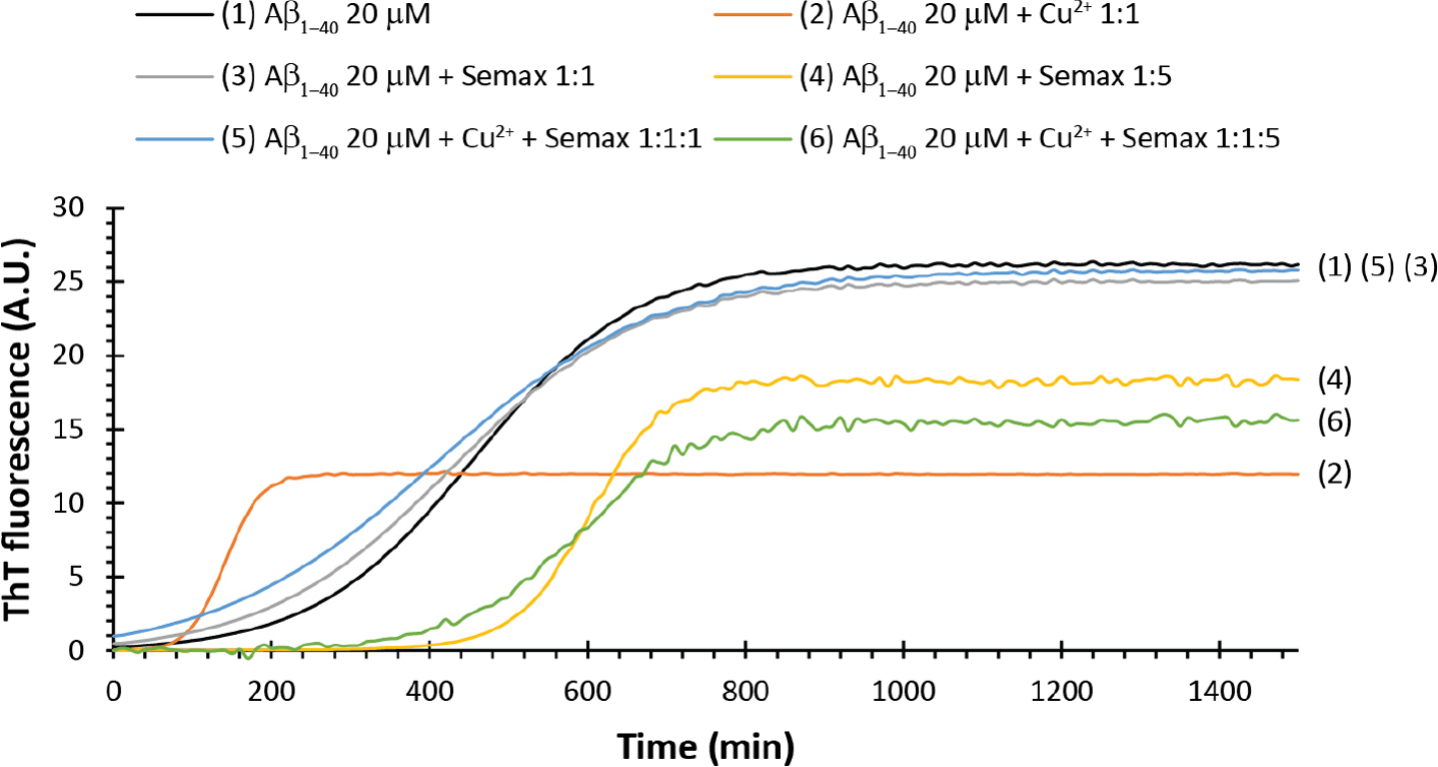
Thioflavin T assay in the MOPS buffer. ThT traces of samples containing Aβ_1–40_ 20 μM alone (black curve) and in the presence of Cu^**2**+^ 20 μM (orange curve), Semax 20 μM (gray curve), Semax 100 μM (yellow curve), Cu^**2**+^ 20 μM/Semax 20 μM (blue curve), and Cu^**2**+^ 20 μM/Semax 100 μM (green curve). All the experiments were performed at 37 °C in the 10 mM MOPS buffer and 100 mM NaCl at pH 7.4. All traces are the average of three independent experiments.

Aβ_1–40_ undergoes fibrillation in the buffer solution, showing a classical sigmoidal curve of Thioflavin T (ThT) fluorescence (Figure 1, black line). The ThT kinetic curve is characterized by a lag time (*t*_lag_, the time in which ThT fluorescence reaches 5% of its maximum), dominated by the formation of oligomeric species not detectable by ThT, followed by an elongation phase, in which pre-fibrillar structures elongate to fiber, normally characterized by a time to half (*t*_1/2_, the time in which the ThT fluorescence reaches half of its maximum value) and, finally, a plateau region in which the equilibrium is reached and the ThT fluorescence reaches its maximum value *I*_max_ (Figure S1 in the Supporting Information). The ThT kinetic curve may be analyzed by using a sigmoidal curve:
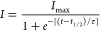
1in which *I*_max_ = maximum fluorescence intensity, *t*_1/2_ = time to half, and τ = elongation time constant. The apparent time constant *k*_app_ is given by 1/τ, and the lag time is defined as *t*_lag_ = *t*_1/2_ – 2τ.^57^

Semax at an equimolar ratio (Figure 1, gray curve) does not significantly perturb any of the kinetic parameters of the Aβ_1–40_ fibrillogenesis process in our condition. Interestingly, the increase of the Semax amount to a ratio of 1:5 (Figure 1, yellow curve) leads to higher *t*_lag_ and *t*_1/2_ (Table 1). Moreover, we also observed a significant decrease in *I*_max_. Thus, Semax shows concentration-dependent anti-aggregating properties. Whether the inhibition of aggregation occurs by the interaction and stabilization of monomeric or oligomeric species is not clear from ThT experiments. However, the longer lag phase followed by faster elongation rate observed for samples containing Semax at higher concentrations (Table 1) suggests the stabilization of oligomers as the more probable mechanism.

**Table 1:** Kinetic Parameters Derived from ThT Experiments Shown in Figure 1^a^

sample	*I*_max_ (A.U.)	*t*_lag_ (min)	*t*_1/2_ (min)	*k*_app_ (min^–1^)	τ (min)
Aβ_1–40_ 20 μM	26.2 ± 0.1	407.2 ± 1.3	457.4 ± 1.2	10·10^–3^ ± 1.0·10^–4^	100.4 ± 6.2
Aβ_1–40_ 20 μM/Cu^2+^ 1:1	11.9 ± 0.1	129.7 ± 0.8	141.0 ± 0.5	4.4·10^–2^ ± 9.5·10^–4^	22.5 ± 9.1
Aβ_1–40_ 20 μM/Semax 1:1	25 ± 0.1	372.8 ± 2.3	430.9 ± 1.5	8.6·10^–3^ ± 9.9·10^–5^	116.2 ± 10.2
Aβ_1–40_ 20 μM/Semax 1:5	18.3 ± 0.1	577.8 ± 3.2	601.5 ± 2.7	2.1·10^–2^ ± 1.0·10^–3^	47.3 ± 7.4
Aβ_1–40_ 20 μM/Cu^2+^/Semax 1:1:1	25.7 ± 0.1	344.6 ± 2.5	412.2 ± 2.0	7.4·10^–3^ ± 9.6·10^–5^	135.1 ± 9.7
Aβ_1–40_ 20 μM/Cu^2+^/Semax 1:1:5	15.5 ± 0.2	544.4 ± 7.3	582.2 ± 6.1	1.3·10^–2^ ± 9.4·10^–4^	75.6 ± 13.1

a ThT curves were fitted by using the equation . Lag time was calculated as *t*_lag_ = *t*_1/2_ – 2τ, and *k*_app_ was given by 1/τ. Results are the average of three independent experiments.

It is known that Cu^2+^ ions interact with Aβ_1–40_ in the 1–16 region,^58,59^ forming a complex and influencing the aggregation process in a concentration-dependent way.^17,60^ The addition of an equimolar amount of Cu^2+^ ions to samples containing Aβ_1–40_ (Figure 1, orange curve) results in a faster fiber formation process with a significant decrease of *t*_lag_ and *t*_1/2_. Moreover, we observe a decrease of the maximum intensity of ThT signal (*I*_max_), as shown in Table 1. A possible explanation is that the presence of Cu^2+^ ion leads to a rapid formation of oligomeric species, which rapidly evolve to mature fibers, with a higher elongation rate than Aβ_1–40_ alone, as evidenced by the increase in the growth rate constant (*k*) and a lower elongation time constant (τ) (Table 1). This behavior is consistent with data reported in the literature; indeed, an equimolar amount of Cu^2+^ is reported to reduce the lag phase^61^ with the appearance of spherical amorphous aggregates and few fibrillar structures.^62^

Semax forms stable complexes with Cu^2+^ ions, with a conditional dissociation constant (^c^Kd) of 1.3·10^–15^ M at pH 7.4.^55^ Since the Aβ_1–40_:Cu^2+^ complex *K*_d_ value is in the nanomolar range,^63−66^ adding Semax to the samples containing Aβ_1–40_ and Cu^2+^ should result in a ThT curve very similar to that observed for Aβ_1–40_ only. As expected, the sample containing Aβ_1–40_/Cu^2+^/Semax 1:1:1 (Figure 1, blue curve) shows a kinetic profile and kinetic parameters very similar to those obtained for Aβ_1–40_ only (Table 1). This evidence confirms that Semax strongly interacts with the Cu^2+^ ion, preventing the formation of Aβ_1–40_:metal complexes. An interesting result is observed when Semax is added in a 5:1 ratio (Figure 1, green curve). We observed an increase in the *t*_lag_ and *t*_1/2_ similar to that for the sample in the absence of Cu^2+^ ions but a further decrease in the *I*_max_ (Table 1), meaning a decrease of the total amount of fiber formed. This evidence suggests that an interaction between the complex Semax:Cu^2+^ and Aβ_1–40_ is occurring.

### Semax Reduces the Abeta Oligomers Levels

Soluble Aβ oligomers are considered the main neurotoxic species in the development of Alzheimer’s disease. Small oligomers can be formed early in AD progression with a strong correlation between their cortical level and cognitive decline.^67^

**Figure 2:**
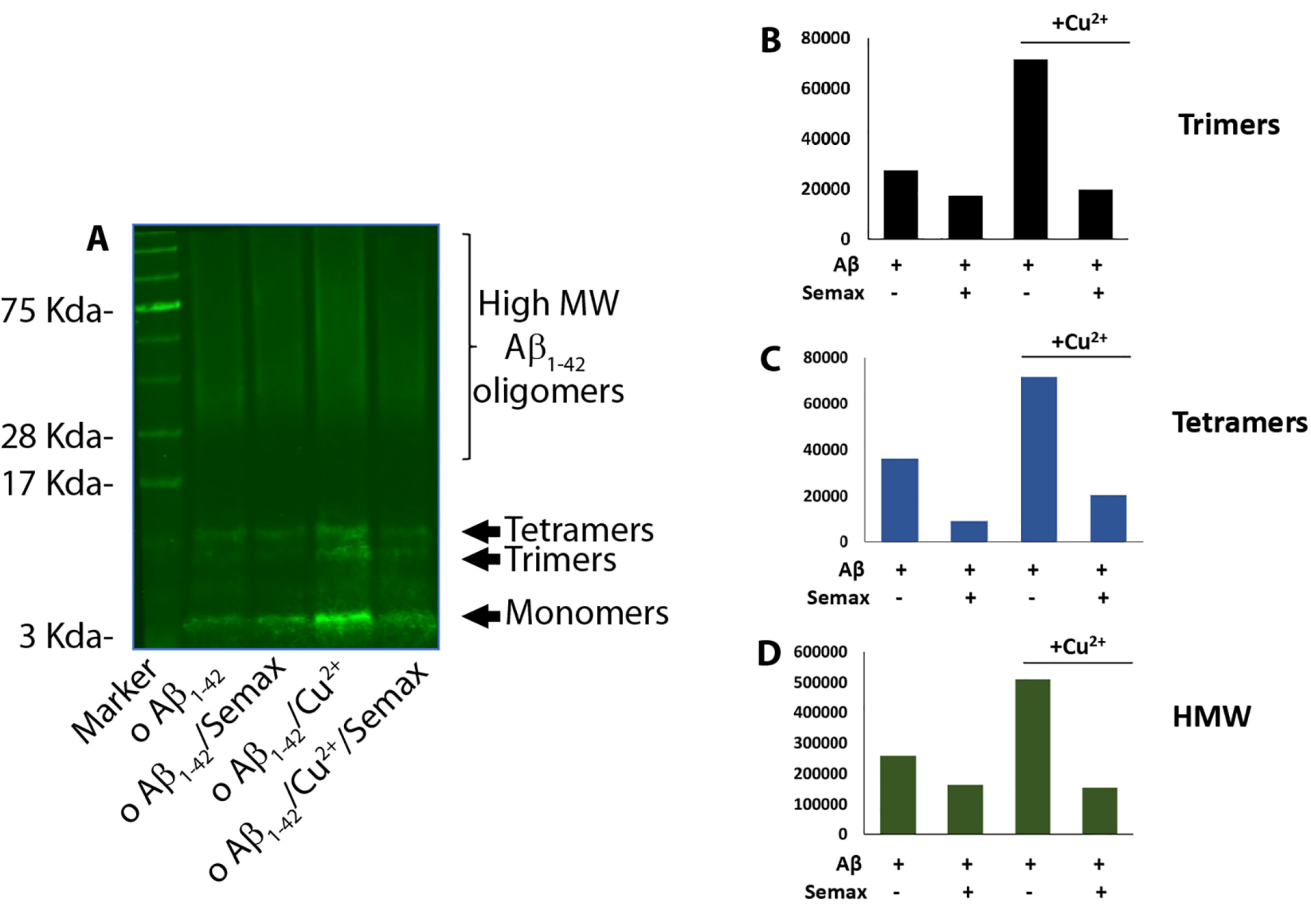
(A) Representative western blot of Aβ_1–42_ oligomers (o Aβ) prepared in the presence of Semax (1:5) and/or copper (1:1). Aβ_1–42_ incubated alone was also prepared as the control. Samples were loaded onto a 4–12% Bis–Tris SDS-PAGE gel and blotted with anti-Aβ N-terminal 1–16 mouse monoclonal antibody 6E10 (1:500). Densitometric analysis of (B) trimeric, (C) tetrameric, and (D) high molecular weight (HMW) bands quantified by the Image Studio software of the Odyssey instrument.

We investigate the ability of Semax to interfere with the early phase of Aβ aggregation by incubating the compound with monomeric forms of amyloid-β 1–42 (Aβ_1−42_) for 48 h at low temperature and under gentle rotation to slow the rate of reaction (Figure 2). In this condition, Aβ_1–42_ is known to oligomerize, forming species with a typical electrophoresis pattern of low molecular weight (monomers, dimers, and tetramers) and high molecular weight bands ranging from 50 to 200 kDa, often appearing as a smear. We used the N-terminus-specific 6E10 Aβ_1–42_ antibody to detect different sizes of aggregated species.

We found that the presence of Semax and/or Cu^2+^ differently affected the normal oligomerization of Aβ_1–42_. To quantify the distribution of oligomeric species produced, we measured the signals of trimeric, tetrameric, and high molecular weight bands by densitometric analysis. We found that co-incubation of Aβ_1–42_ with Semax, at molar ratio of 1:5, produced a decrease of signal intensity of either low and higher molecular weights bands. A slighter increase was seen in the same molecular weight size in the presence of copper 1:1. Interestingly, the presence of Semax in the Aβ/copper sample was reported to lower the level of all the bands corresponding to Aβ oligomeric species.

### Semax Prevents Aβ_1–40_ Interaction with the Hydrophobic Core of the Model Membrane in the Presence of Cu^2+^

The lipidic membrane plays a critical role in the aggregation of amyloidogenic protein.^68−70^ It is known that the interaction with the hydrophobic core and/or the surface of the membrane could catalyze fibril formation.^71^ Typically inneuronal cells, the ratio between zwitterionic and negatively charged phospholipids headgroup is 3:1. To resemble this ratio, we performed differential scanning calorimetry (DSC) to study the interaction of Aβ_1–40_ in the presence of model membranes composed of large unilamellar vesicles (LUV) DMPC:DMPS 3:1. DSC is a useful tool that allows the study of the topology and the amount of peptide membrane interaction^72,73^ by evaluating the enthalpy (Δ*H*) and the transition temperature (*T*) associated with the gel/liquid crystal transition of the phospholipid bilayer.^74^ The thermogram of DMPC:DMPS 3:1 LUV shows a single peak (Figure S2, black curve) centered at 27.5 °C and a Δ*H* of 29.3 kJ mol^–1^ (Table S1). The addition of Aβ_1–40_ induces a remodulation of the spatial distribution of the lipid (Figure S3A), which is also a function of the temporal evolution of samples (Figure S4A–D). Phase segregation has been observed several times in the literature and depends on the nature of the interaction of a protein or a peptide with the phospholipid bilayer.^72,75−77^ In our samples, three distinct peaks appear in the thermogram (Figure S3A). The first, well resolved, at the lower temperature could be assigned to the region of the membrane richer in zwitterionic DMPC. The second and the third ones, which convolute each other, represent regions of the bilayer richer in negatively charged DMPS. Deconvolution of peaks allows one to estimate the region of interaction of the protein (Figure S3B).

**Figure 3:**
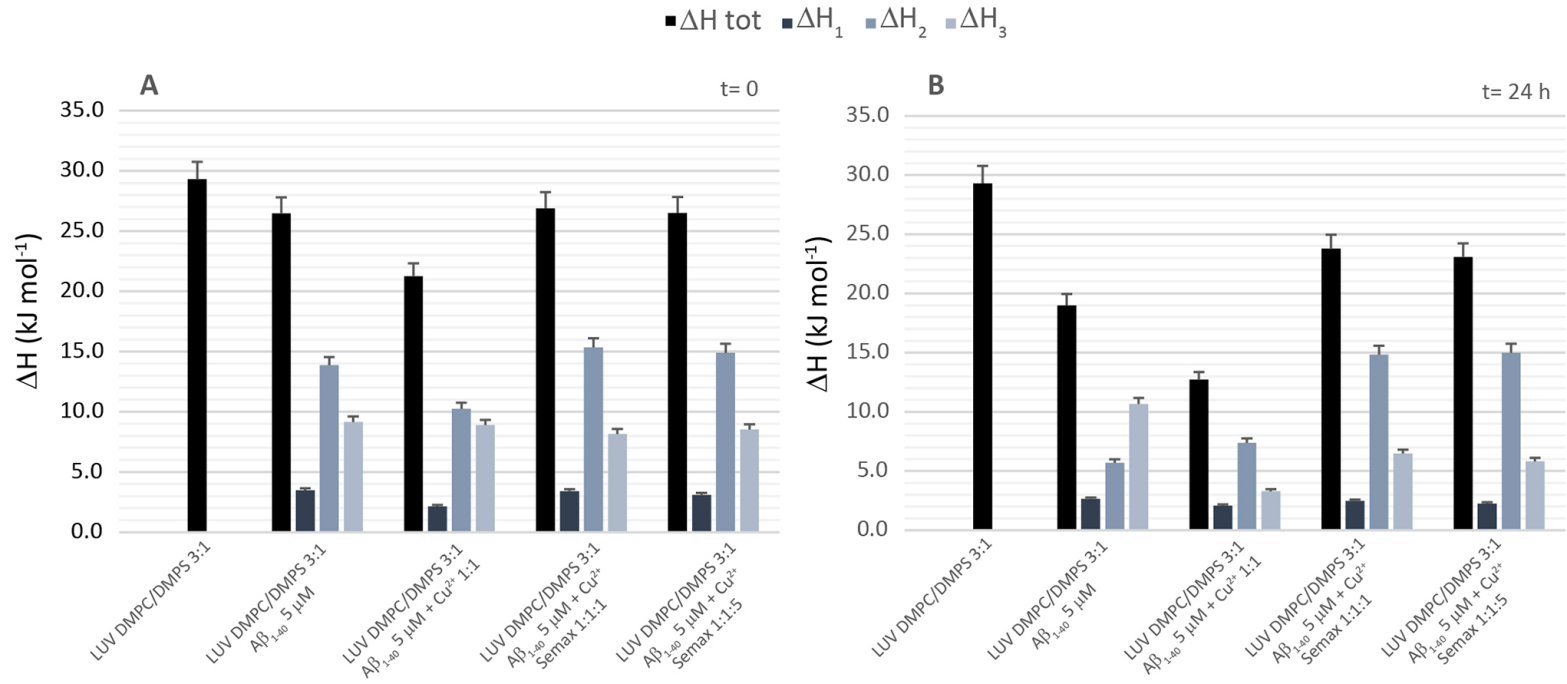
Comparison of enthalpy contribution. Contribution of Δ*H* of each peak for LUV DMPC:DMPS in the presence of Aβ_1–40_ 5 μM, Aβ_1–40_ 5 μM + Cu^**2**+^ 5 μM, Aβ_1–40_ 5 μM + Cu^**2**+^ 5 μM + Semax 5 μM, and Aβ_1–40_ 5 μM + Cu^**2**+^ 5 μM + Semax 25 μM at (A) time 0 and (B) 24 h.

At time 0, the main species in the solution are monomeric Aβ. The thermogram of the sample containing Aβ_1–40_ alone (Figure S2A, red curve) shows a decrease in the overall Δ*H* (Table S2), which is the sum of three contributions as explained before. As expected, the presence of Cu^2+^ ions (Figure S2A, green curve) enhances the interaction of Aβ_1–40_ with the hydrophobic core of the bilayer as evidenced by the further decrease of the Δ*H*_tot_. By comparing the Δ*H* of each peak, it appears that the interaction mainly involves the region of the membrane with a lipidic composition similar to that of the reference (3:1), with a significant decrease in Δ*H*_2,_ while Δ*H*_1_ and Δ*H*_3_, associated with a region richer in zwitterionic lipid and negatively charged lipid, respectively, remain substantially unaltered (Figure 3A and Table S1). As expected, the addition of Semax, at both 1:1 and 1:5 ratios (Figure 3A, yellow and blue curves), results in thermodynamic parameters (Table S1) very similar to those of the sample containing only Aβ_1–40_. This result matches very well with what was observed in ThT experiments, and it is mostly due to the formation of the Semax/Cu^2+^ complex that is more stable than the Aβ_1–40_/Cu^2+^ complex.

Letting the sample evolve, after 6 h, the main species in the solution are oligomeric or pre-fibrillar species. The thermogram of the sample containing Aβ_1–40_ (Figure S2B, red curve) shows a further decrease of Δ*H*_tot_, meaning that the protein is continuing to insert through the lipid hydrophobic tails. In particular, we observed a further decrease of Δ*H*_2_ (Table S2). The sample containing Cu^2+^ (Figure S2B, green curve) also shows a further decrease of Δ*H*_tot_ and, in particular, Δ*H*_2_ (Table S2), stronger than that of Aβ_1–40_ alone (Figure S5). Interestingly, the addition of Semax seems to inhibit the further insertion of prefibrillar species in the hydrophobic core of the bilayer (Figure S2B, yellow and blue curves). We observed a Δ*H*_tot_ higher not only than that of Aβ_1–40_/Cu^2+^ but also than that of Aβ_1–40_ alone (Table S2). By comparing the contribution of each peak (Figure S5), we observed that the main effect, which is concentration dependent, is on the second peak as evidenced by the values of Δ*H*_2_. This suggests the hypothesis that the Semax/Cu^2+^ complex mainly interacts with oligomeric species of Aβ_1–40_ by both retarding their formation and hampering their interaction with the lipidic membrane.

After 24 h, the fiber formation process is completed. The main species in the solution are mature fibers. For each sample, we observed a further significant decrease of the Δ*H*_tot_ (Table S3). This could be explained keeping in mind that Aβ_1–40_ disrupts the integrity of the model membrane through a two-step mechanism: the first step is the formation of pores, and the second step is correlated with the elongation of the fibers on the membrane surface and the disruption through a detergent-like mechanism.^72^ The presence of Cu^2+^ (Figure S2, green curve) dramatically decreased Δ*H*_tot_. Comparing the contribution of single peaks (Figure 3B), it is evident that the presence of Cu^2+^ decreases Δ*H*_3_ significantly_,_ meaning that it enhances the interaction with the region of the membrane richer in negatively charge phospholipids. Interestingly, the presence of Semax (Figure S2, yellow and blue curves), independent of the concentration, hampers the interaction of mature fibrils with the phospholipidic bilayer. In particular, we observed higher values of Δ*H*_2_ than those of samples containing Aβ_1–40_ alone or in the presence of Cu^2+^.

The overall results suggest that the presence of Semax modifies the interaction of Aβ_1–40_ with the model membrane not only by sequestering Cu^2+^ but by interacting most probably with the oligomers.

### Semax Strongly Inhibits Fiber Formation in the Presence of bTLE Model Membranes

**Figure 4:**
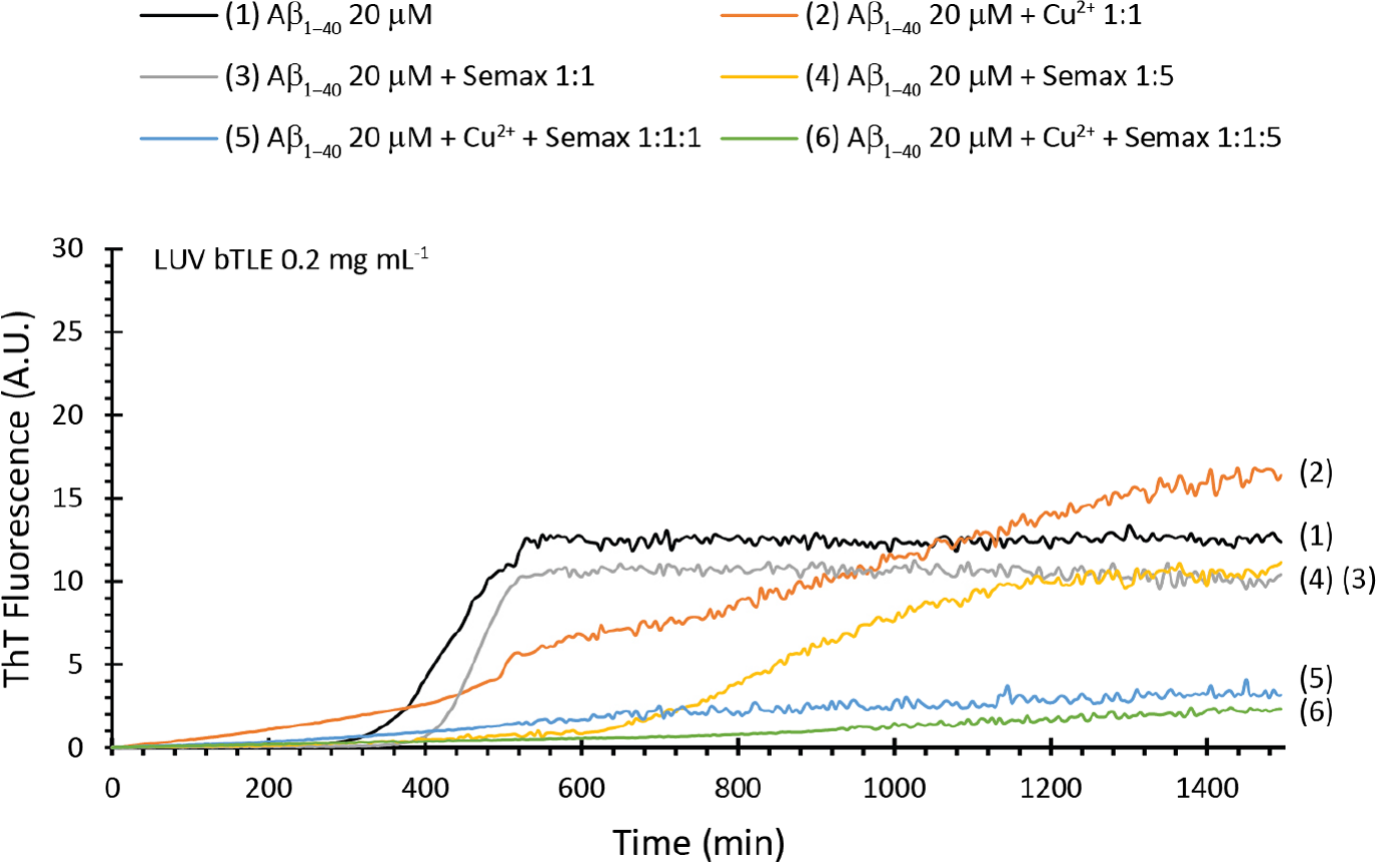
Thioflavin T assay in the presence of bTLE LUVs. ThT traces of samples containing LUV bTLE 0.2 mg mL^–**1**^ and Aβ_1–40_ 20 μM alone (black curve) and in the presence of Cu^**2**+^ 20 μM (orange curve), Semax 20 μM (gray curve), Semax 100 μM (yellow curve), Cu^**2**+^ 20 μM/Semax 20 μM (blue curve), and Cu^**2**+^ 20 μM/Semax 100 μM (green curve). All the experiments were performed at 37 °C in aCSF at pH 7.4. All traces are the average of three independent experiments.

Membrane composition as well as the environment plays a critical role in the aggregation process of Aβ. For this reason, we performed experiments in the presence of LUV composed of the brain total lipid extract (bTLE), which resembles neuron lipid composition, dispersed in the artificial cerebrospinal fluid (aCSF) at pH 7.4. Aβ_1–40_ in this condition shows the typical sigmoidal curve (Figure 4, black curve) characterized by a *t*_1/2_ of ∼239 min and an *I*_max_ of ∼12.7 (Table 2). Semax, added to our samples, shows an interesting concentration-dependent effect. When added in a stoichiometric ratio (Figure 4, gray curve), it does not perturb significantly the kinetics of fiber formation. We observed a small increase in the lag phase and a small decrease of the total mass of fiber formed (Table 2). When Semax is added in a 5:1 ratio (Figure 4, yellow curve), it significantly increases the lag phase (Table 2) but does not reduce the total amount of fiber formed, which is identical to that observed at lower concentrations. This evidence suggests a concentration-dependent interaction of Semax with Aβ_1–40_ oligomers.

**Table 2:** Kinetic Parameters Derived from ThT Experiments Shown in Figure 4^a^

sample	*I*_max_ (A.U.)	*t*_lag_ (min)	*t*_1/2_ (min)	*k* (min^–1^)	τ (min)
Aβ_1–40_ 20 μM	12.7 ± 0.2	239.0 ± 2.5	439.0 ± 2.8	2.5·10^–2^ ± 1.2·10^–3^	40.0 ± 2.3
Aβ_1–40_ 20 μM/Cu^2+^ 1:1	21.5 ± 0.1	814,5 ± 3.2	986.9 ± 3.5	2.8·10^–3^ ± 1.0·10^–4^	357.1 ± 5.2
Aβ_1–40_ 20 μM/Semax 1:1	10.5 ± 0.5	353.3 ± 5.6	462.0 ± 6.6	4.6·10^–2^ ± 1.8·10^–3^	21.7 ± 3.6
Aβ_1–40_ 20 μM/Semax 1:5	10.5 ± 0.5	806.6 ± 4.3	865.4 ± 4.1	8.5·10^–2^ ± 2.2·10^–3^	11.8 ± 5.1
Aβ_1–40_ 20 μM/Cu^2+^/Semax 1:1:1	3.7 ± 0.2	554.7 ± 2.1	706.2 ± 1.8	3.3·10^–3^ ± 1.2·10^–4^	303.0 ± 6.2
Aβ_1–40_ 20 μM/Cu^2+^/Semax 1:1:5	5.8 ± 0.1	1407.7 ± 10.6	1635.0 ± 12.4	2.2·10^–3^ ± 2.3·10^–4^	454.5 ± 13.5

a ThT curves were fitted by using the equation . Lag time was calculated as *t*_lag_ = *t*_1/2_ – 2τ, and *k*_app_ was given by 1/τ. Results are the average of three independent experiments.

The presence of Cu^2+^ 1:1 alone (Figure 4, orange curve) totally modifies the kinetics of fiber formation. It is evident that the presence of metal ions retards fiber formation with a significant increase of lag phase, reduces the elongation rate, but induces the formation of more fiber at the end of the process (Table 2). Interestingly, the presence of Semax in samples containing Cu^2+^ ions seems to inhibit, almost completely, the Aβ_1–40_ fiber formation (Table 2) at both lower (Figure 4, blue curve) and higher (Figure 4, green curve) concentrations. This result was unexpected since a simple Semax/Cu^2+^ complex formation should result in a behavior similar to that observed in the absence of metal ions or, at least, for the sample containing a higher amount of Semax, to that observed in the presence of Semax only. In our experimental condition, Semax was added after the Aβ_1–40_/Cu^2+^ complex formation; thus, it is reasonable to think that Semax not only subtracts Cu^2+^ from the Aβ_1–40_/Cu^2+^ complex but is able to interact and stabilize oligomers formed starting from these complexes. Although beyond the purpose of this work, we try to prove this hypothesis by letting the Semax/Cu^2+^ complex interact with Aβ_1–40_ (Figure S6). In these conditions, what we observed is a completely different result, with the kinetics of fiber formation more similar to those observed for the sample containing Aβ_1–40_ only. This result strengthens our hypothesis, which in any case needs more in-depth studies to be confirmed.

### Semax Prevents Membrane Disruption Induced by Aβ_1–40_

**Figure 5:**
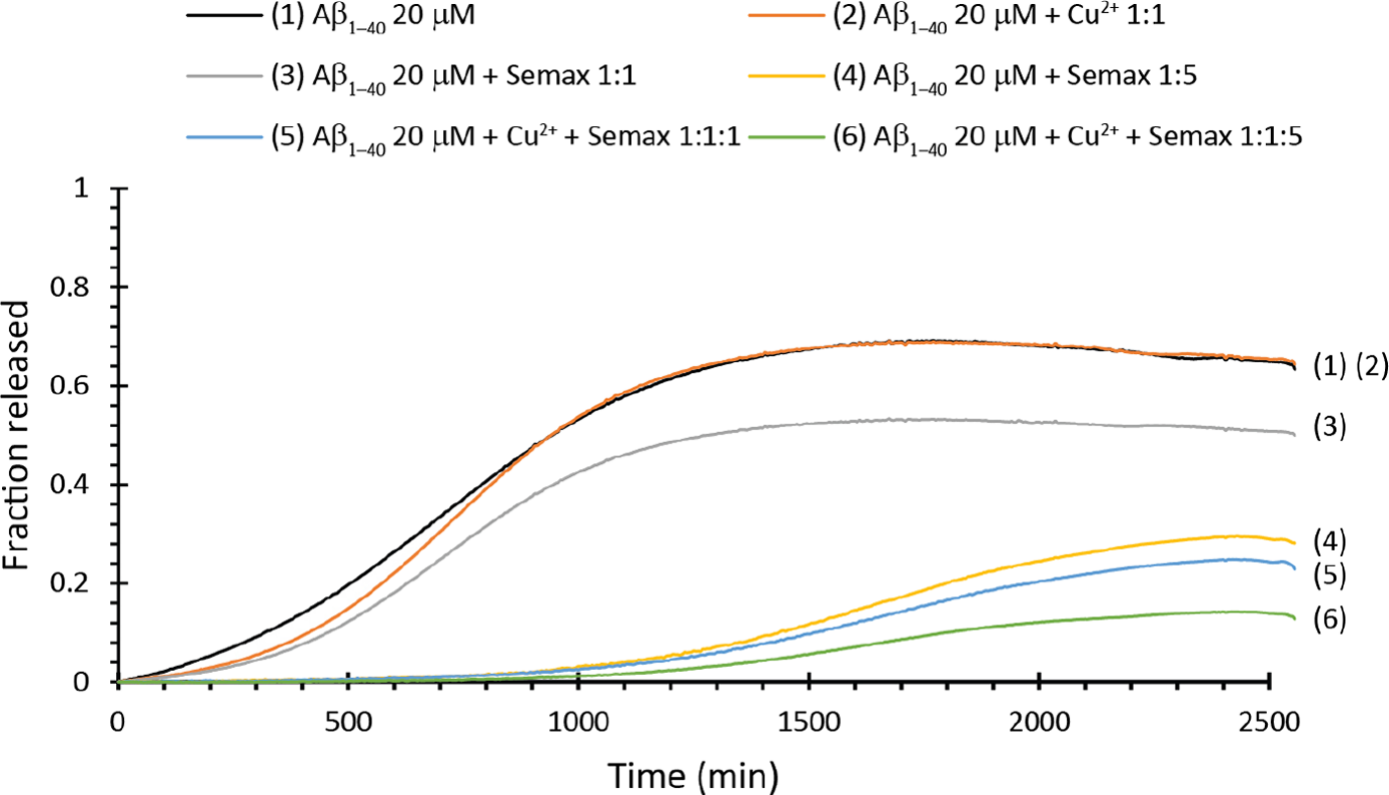
Dye leakage assay in the presence of bTLE LUVs. Dye release for samples containing LUV bTLE 0.2 mg mL^–1^ and Aβ_1–40_ 20 μM alone (black curve) and in the presence of Cu^**2**+^ 20 μM (orange curve), Semax 20 μM (gray curve), Semax 100 μM (yellow curve), Cu^**2**+^ 20 μM/Semax 20 μM (blue curve), and Cu^**2**+^ 20 μM/Semax 100 μM (green curve). All the experiments were performed at 37 °C in aCSF at pH 7.4. All traces are the average of three independent experiments.

The mechanism of model membrane disruption induced by Aβ_1–40_ could be studied by using a dye leakage experiment. It was demonstrated that Aβ_1–40_, as well as other amyloidogenic proteins, is able to disrupt the model membrane with a two-step mechanism.^78−81^ In particular, the second step of membrane disruption is correlated with the elongation of the fibers on the membrane surface through a detergent-like mechanism.^78^ Here we performed a dye leakage assay on samples containing bTLE LUV filled with 5(6)-carboxyfluorescein. Aβ_1–40_ alone (Figure 5, black curve) shows a leakage of ∼68% with a *t*_1/2_ of ∼688 min (Table 3), a value comparable with ThT *t*_1/2_ in the same condition. Interestingly, Semax reduces in a concentration-dependent way the total amount of membranes leaked. In particular, the total amount of membrane disrupted is ∼52% when added in a 1:1 ratio (Figure 5, gray curve) and ∼31% when added in 5:1 ratio (Figure 5, yellow curve). These results match very well with those observed with the ThT assay, also in terms of *t*_1/2_ (Table 3), which are comparable to those of fiber formation. Semax, consistent with what was observed in ThT experiments, seems to protect the membrane from disruption induced by Aβ_1–40_.

**Table 3:** Parameters Derived from Dye Leakage Experiments Shown in Figure 5^a^

sample	*F*_max_	*t*_1/2_ (min)
Aβ_1–40_ 20 μM	0.68 ± 0.05	688 ± 2
Aβ_1–40_ 20 μM/Cu^2+^ 1:1	0.68 ± 0.06	733 ± 3
Aβ_1–40_ 20 μM/Semax 1:1	0.52 ± 0.05	712 ± 2
Aβ_1–40_ 20 μM/Semax 1:5	0.31 ± 0.09	1631 ± 2
Aβ_1–40_ 20 μM/Cu^2+^/Semax 1:1:1	0.26 ± 0.07	1643 ± 3
Aβ_1–40_ 20 μM/Cu^2+^/Semax 1:1:5	0.15 ± 0.03	1604 ± 2

a Dye leakage curves were fitted by using the Boltzmann curve. Results are the average of three independent experiments.

The presence of Cu^2+^ (Figure 5, orange curve) does not significantly modify membrane disruption (Table 3). But, consistent with ThT experiments, the addition of Semax at both lower (Figure 5, blue curve) and higher (Figure 5, green curve) concentrations significantly reduces the total amount of membrane disrupted (Table 3) to ∼26 and ∼15%, respectively, with *t*_1/2_ comparable to that observed in ThT experiments. Thus, the whole results strengthen the hypothesis that Semax could interfere with oligomeric species formed by Aβ_1–40_, which in turn are responsible for the kinetics of fiber formation and the consequent disruption of model membranes.

### Semax Rescues Abeta and Cu-Associated Abeta Toxicity

**Figure 6:**
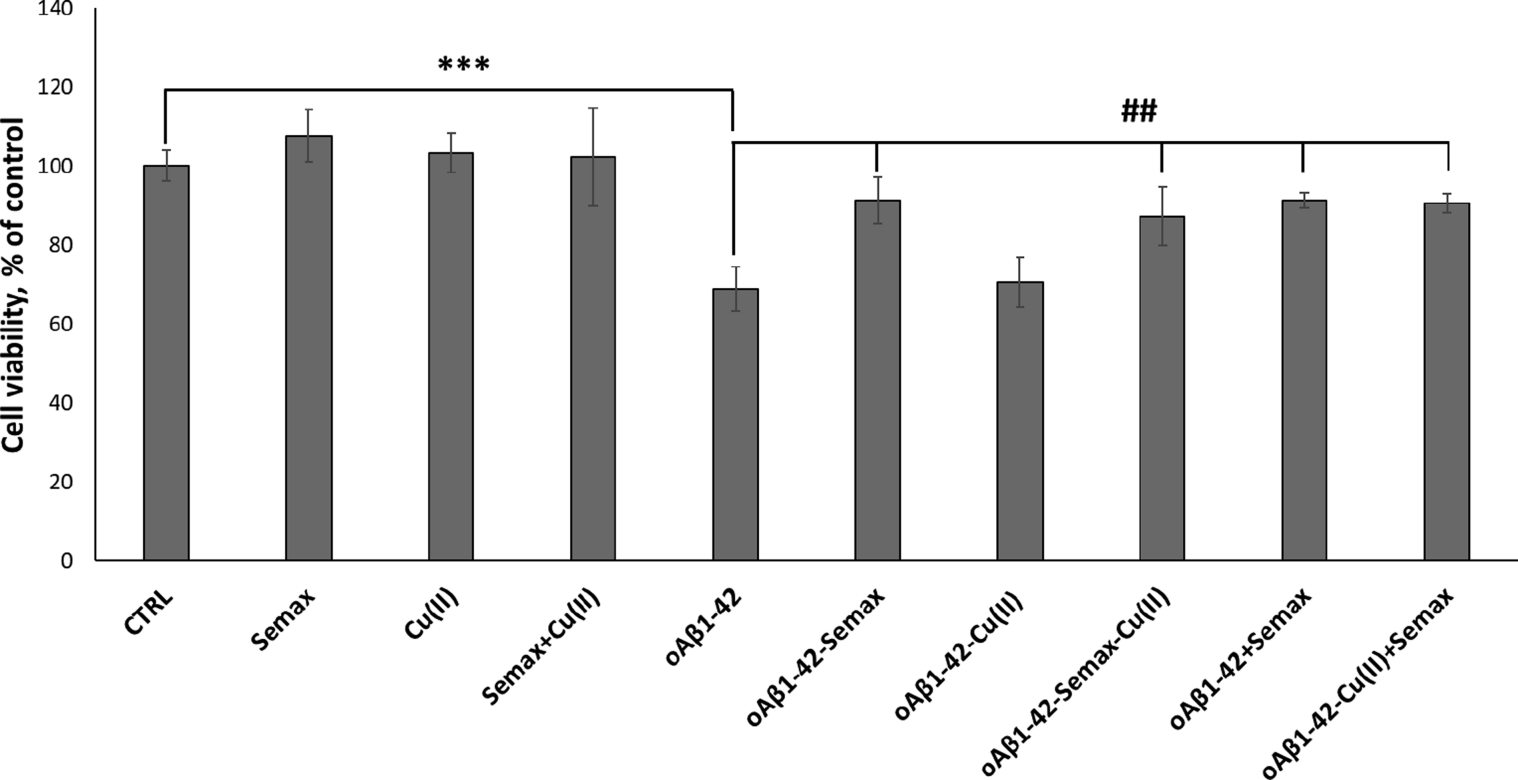
Viability of d-SH-SY5Y cells after 48 h of incubation with 5 μM differently pre-incubated Aβ_1–42_ oligomers with/without Semax and Cu^**2**+^. Results are presented as mean ± SD with *n* = 8 for each condition (***p ≤ 0.001with respect to untreated control; ##*p* ≤ 0.01 with respect to Aβ_1–42_ treatment).

It has previously been shown that Aβ_1–42_ oligomeric aggregates form insoluble amyloid deposits in the brain and hippocampus and exhibit neurotoxic effects in in vitro and in vivo models, leading to the cognitive dysfunction prevalent in the pathogenesis of AD.^82,83^ Thus, to assess the cytotoxicity of the oligomeric species, the effect of Aβ_1–42_ at different preparations of oligomerization on the viability of d-SH-SY5Y cells was investigated via an MTT assay (Figure 6). Cells were treated for 48 h with Aβ_1–42_ that was pre-incubated for 48 h with/without Semax or Cu^2+^ (see Materials and Methods). In agreement with previously reported results,^33^ we found a decrease of d-SH-SY5Y cell viability after 48 h treatment with 5 μM Aβ_1–42_ oligomers. According to the data observed in the above experiments showing Semax protection against Aβ_1–42_ oligomer stress, the MTT assay reveals that the presence of exogenous Aβ_1–42_ oligomers decreases cell viability up to 32%. Noteworthily, the Aβ_1–42_ oligomers prepared in the presence of Semax or/and Cu^2+^ (see Materials and Methods) show a significant effect on cell viability, reducing toxicity by more than 22% in the case of Semax alone and 20% in the case of Semax with Cu^2+^. Another set of experiments was performed to understand the effect of Semax on the toxicity of already formed Aβ_1–42_ oligomers. First, we prepared oligomers and then we added Semax with/without copper to the cells. As Figure 6 shows, just as in the case of oligomers preincubated for 48 h in the presence of Semax or Semax-Cu(II) and then added to the d-SH-SY5Y cells, the Semax in this case also protects the cells from the toxicity of the already pre-formed oligomers, reducing Aβ_1–42_-induced cell toxicity by up to 90% in both the case with and that without copper.

## Conclusions

In summary, we performed a series of biophysical experiments that show that the heptapeptide ACTH(4–7)-PGP (Semax) is able to inhibit, in the absence of the model membrane, Aβ fiber formation. In particular, we showed that in the presence of Cu^2+^ ions, Semax is able not only to sequester metal ions, inhibiting Aβ:Cu^2+^ complex formation, but also to interact, most probably, with Aβ:Cu^2+^ oligomers, retarding the formation of protofibrillar and fibrillar species. DSC results showed that the heptapeptide modulates the interaction of Aβ:Cu^2+^ with the hydrophobic core of the model membrane, preventing or retarding the insertion of prefibrillar species in the hydrophobic core of the model membrane. This finding is corroborated by the evidence that in the presence of the model membrane of bTLE, Semax could by itself inhibit fiber formation but exerts the maximum of its anti-aggregating properties in the presence of Cu^2+^ ions. This suggests that the heptapeptide interacts differently with monomeric/oligomeric species of Aβ and Aβ:Cu^2+^ complexes. This different behavior is confirmed by dye leakage experiments that, according to the ThT assay, showed a protective role of Semax. Finally, the MTT assay revealed that Semax or the Semax:Cu^2+^ complex protects cells from the toxicity of Aβ_1–42_ oligomers. More in-depth experiments should be done especially to investigate the effect of Semax on ROS production, which is known to increase as an effect of Aβ:Cu^2+^ interaction.

## Material and Methods

### Materials

Aβ_1–40_ and Aβ_1−42_ was purchased from Bachem (Bubendorf, Switzerland) with a purity ≥95%. Semax (H-Met-Glu-His-Phe-Pro-Gly-Pro-OH) was purchased from Sigma Aldrich (St. Louis, MO, USA) with a purity ≥98%. 1,2-Dimyristoyl-*sn*-glycero-3-phosphocholine (DMPC), 1,2-dimyristoyl-*sn*-glycero-3-phospho-l-serine (DMPS), and brain total lipid extract (bTLE) were purchased from Avanti Polar Lipids (Alabaster, AL, USA). Thioflavin T, 6-carboxyfluorescein, NaCl, NaHCO_3_, KCl, NaH_2_PO_4_, MgCl_2_, glucose, CaCl_2_, and all other salts were purchased from Sigma-Aldrich (St. Louis, MO, USA).

### Preparation of the Artificial Cerebrospinal Fluid

The buffer used for some experiments is the artificial cerebrospinal fluid (aCSF). aCSF was prepared by dissolving in Millipore water 119 mM NaCl, 26.2 mM NaHCO_3_, 2.5 mM KCl, 1 mM NaH_2_PO_4_, 1.3 mM MgCl_2_, 10 mM glucose, and 2.5 mM CaCl_2_. The final pH was 7.4. The buffer was stored at 4 °C and was stable for at least 3 weeks.

### Peptide Preparation

To prevent the presence of any preformed aggregates, Aβ_1–40_ was initially dissolved in HFIP at a concentration of 1 mg/mL and then lyophilized overnight. To be used for the experiments, the lyophilized powder was initially dissolved in NH_3_OH 6 M to obtain a stock solution with a final concentration of 250 μM. The concentration of Aβ was determined by using the absorption of tyrosine at λ 280 nm (ε = 1490 M^–1^ cm^–1^). The Semax concentration was calculated by copper UV titration. According to equilibrium studies,^55^ Semax forms a single complex species (100%), the [CuLH–2] ^2–^, with the donor atoms around copper arranged in a 4 N planar coordination mode, at pH 7.4 with a conditional dissociation constant (^c^Kd) of 1.3 × 10^–15^ M. The Cu(NO_3_)_2_ stock solution was prepared and standardized with ethylenediaminetetraacetic acid, as reported by Flashka.^84^ Each stock solution was used immediately after preparation by diluting in the opportune buffer solution to reach the concentration needed for experiments.

### LUV Preparation

We used large unilamellar vesicles (LUVs) composed of a mixture of zwitterionic and negatively charged lipids (DMPC and DMPS in 3:1 ratio) or the brain total lipid extract (bTLE). Model membranes were prepared as described elsewhere.^85^ Briefly, appropriate aliquots of lipid stock solutions in chloroform were dried by using a stream of dry nitrogen and evaporated overnight under high vacuum to dryness in a round-bottomed flask. Initially, multilamellar vesicles (MLVs) were obtained by hydrating the lipid film with an appropriate amount of buffer (aCSF buffer 10 mM (pH 7.4) or MOPS buffer 10 mM and 100 mM NaCl (pH 7.4)) and dispersing by vigorous stirring. MLVs were then extruded through polycarbonate filters (pore size = 100 nm, Nuclepore, Pleasanton, CA) mounted in a mini-extruder (Avestin, Ottawa, Canada) fitted with two 0.5 mL Hamilton gastight syringes (Hamilton, Reno, NV) to obtain LUVs. Samples were typically subjected to 23 passes through two filters in tandem and as recommended elsewhere.^86^

### ThT Measurements

The kinetics of aβ fiber formation were measured using the Thioflavin T (ThT) assay. Samples were prepared by diluting, in MOPS buffer or in a solution containing bTLE LUVs, the aβ stock solution to reach the final concentration. Semax and/or copper were added from a stock solution to the indicated concentration. Thioflavin T was then added to a final concentration of 40 μM. Experiments were carried out in Corning 96-well non-binding surface plates. Time traces were recorded using a Varioskan (ThermoFisher, Waltham, MA) plate reader using a λ_ex_ of 440 nm and a λ_em_ of 485 nm at 37 °C, shaking the samples for 10 s before each read. All ThT curves represent the average of three independent experiments.

### Dye Leakage Measurements

Membrane leakage experiments were performed by measuring the leakage of 6-carboxyfluorescein dye from LUVs. Dye-filled bTLE LUVs were prepared by hydrating the dry lipid film with the buffer solution containing 6-carboxyfluorescein (80 mM 6-carboxyfluorescein, pH 7.4) according to the procedure described above. Non-encapsulated 6-carboxyfluorescein was removed by eluting the solution containing LUVs through a Sephadex G50 gel exclusion column (Sigma-Aldrich, St. Louis, MO) using the buffer solution. The final concentration of lipids was checked by using the Stewart assay as described elsewhere.^87^ Membrane damage was quantified by the increase in fluorescence emission intensity of 6-carboxyfluorescein due to its dilution (de-quenching) in the buffer as a result of the membrane leakage. Time traces were recorded in Corning 96-well non-binding surface plates using a Varioskan (ThermoFisher, Waltham, MA) plate reader using a λ_ecc_ of 490 nm and a λ_em_ of 510 nm at 37 °C, shaking the samples for 10 s before each read. All curves represent the average of three independent experiments.

### DSC Experiments

DSC runs of DMPC/DMPS 3:1 LUVs were carried out on a Nano-DSC (TA Instruments, Inc., New Castle, DE) apparatus. Lipid samples were degassed by vacuum and then heated from 20 to 40 °C at a scan rate of 1 °C min^–1^. A lipid/peptide ratio of 20:1 was used in all the experiments. An extra external pressure of about 3 atm was applied on the solution to prevent the formation of bubbles during heating. The MOPS buffer solution 10 mM and 100 mM NaCl (pH 7.4) were used as reference. Heat capacity curves (Cp) were obtained by subtracting the buffer–buffer baseline from raw DSC data. All DSC runs were performed immediately after the preparation of samples and after 6 and 24 h to observe if kinetic effects are present.

### Anti-oligomerization Assay

Aβ_1–__42_ oligomers were prepared as previously described^11^ from synthetic Aβ_1__–__42_ following the protocol of monomerization. Aβ_1__–__42_ (0.3 mg) was firstly dissolved in 5 mM DMSO. A solution of 100 μM Aβ_1–42_ in ice-cold DMEM F-12 without phenol red was prepared and allowed to oligomerize for 48 h at 4 °C according to the Lambert protocol^88^ with some modifications as previously described.^11^ To evaluate the ability of SEMAX and/or copper to interfere with Aβ oligomer formation, samples of Aβ_1–42_ were incubated in the presence or absence of each compound (Aβ/ligand ratios of 1:5 and 1:1 respectively). After 48 h incubation, Aβ/ligand compounds were analyzed for their content of Aβ oligomers.

### Western Blot Analysis

After incubation, the amount and size of Aβ aggregates were determined by western blot analysis. Unheated samples (25 μL) were loaded onto precast Bis–Tris gel (Bolt 4–12%, Life Technologies) with 2-morpholin-4-ylethanesulfonic acid (MES). Samples were transferred onto a nitrocellulose membrane (0.2 mm, Hybond ECL, Amersham Italia) by a wet transfer unit (Mini Blot Module, Life Technologies). Membranes were blocked in the Odyssey blocking buffer (Li-COR Biosciences) and incubated at 4 °C overnight with the mouse monoclonal anti-amyloid-β antibody against N-terminal 1–16 peptide (1:1000) (6E10, Covance). Goat anti-mouse secondary antibodies labeled with IR dye 800 (1:25,000) were used at RT for 45 min. Hybridization signals were detected with the Odyssey CLx Infrared Imaging System (LI-COR Biosciences Lincoln, Nebraska, USA), and densitometric analysis was performed by the use of the Image Studio software.

### Cell Maintenance and Treatment Protocol

The human neuroblastoma cell line (SH-SY5Y) was cultivated in the full medium, i.e., DMEM F-12 supplemented with 10% FBS, 2 mM l-glutamine, and 100 μg mL^–1^ streptomycin, in tissue-culture treated Corning flasks (Sigma-Aldrich). For differentiation of SH-SY5Y (d-SH-SY5Y), cells were seeded at a density of 1.5 × 10^4^ cells/well and neuronal differentiation was induced by treatment for 7 days with 10 μM retinoic acid (RA) in DMEM F-12 medium supplemented with 0.5% of FBS. The cell culture was grown in tissue-culture treated Corning flasks (Sigma-Aldrich) in a humidified atmosphere (5% CO_2_) at 37 °C (HeraCell 150C incubator, Heraeus, Hanau, Germany).

### MTT Assay

Aβ_1–42_ peptide oligomers were prepared as described in ″Anti-oligomerization Assay″ with/without Semax and copper. d-SH-SY5Y cell viability was tested by incubation with 5 μM Aβ_1–42_ samples for 48 h in DMEM supplemented with 0.5% of FBS without RA. The viable cells were quantified by the reaction with MTT. After 90 min, the reaction was stopped by adding DMSO, and absorbance was measured at 570 nm (Varioskan Flash Spectral Scanning Multimode Readers, Thermo Scientific, Waltham, MA, USA); the results were expressed as % of viable cells. The experiments were repeated with *n* = 8, and results were expressed as mean ± SD.

## Supporting Information Available

DSC thermograms, thermodynamic data and comparison, and ThT in the presence of model membranes (PDF)
