# Supplementary material for: Semax, a Synthetic Regulatory Peptide, Affects Copper-Induced Abeta Aggregation and Amyloid Formation in Artificial Membrane Models
Source: ACS Chem Neurosci. 2022 Jan 26;13(4):486–96. doi: 10.1021/acschemneuro.1c00707 (PMC8855339; doi:10.1021/acschemneuro.1c00707)
Supplement: Supplementary file 1 — cn1c00707_si_001.pdf [file cn1c00707_si_001.pdf]

Supporting Information

Semax, a synthetic regulatory peptide, affects copper-induced Abeta aggregation and amyloid formation in artificial membrane models

Michele F.M. Sciacca,Irina Naletova, Maria Laura Giuffrida, Francesco Attanasio**

Consiglio Nazionale delle Ricerche, Istituto di Cristallografia, Via Paolo Gaifami, 18, Catania, Italy

**Corresponding Author:*

Michele Francesco Maria Sciacca: michelefrancescomaria.sciacca@cnr.it

Francesco Attanasio: francesco.attanasio@cnr.it

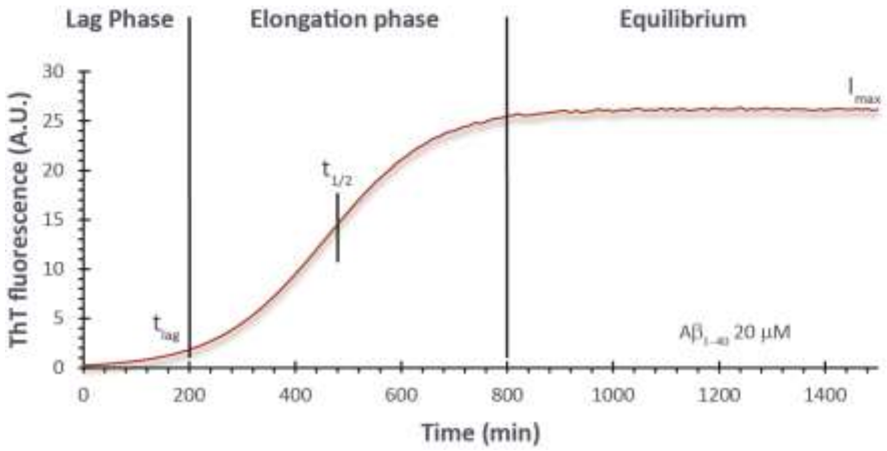

Figure S1. Classical sigmoidal ThT fluorescence curve. Lag phase is the time the system needs to form aggregation nuclei through the formaton of oligomeric species. It is characterized by t_{lag} which is by definition the time the fluorescence needs to reach 5% of the maximum value. Elongation phase is the time the prefibrillar and fibrillar species elongates by addition of monomers or other nuclei to form mature fiber. It is characterized by $t_{1/2}$ which is by definition the time the fluorescence needs to reach half of the maximum intensity. Equilibrium phase is characterized by I_{max}, the maximum of fluorescence intensity which is proportional to the mass of fiber formed. In this phase the system reaches the equilibrium.

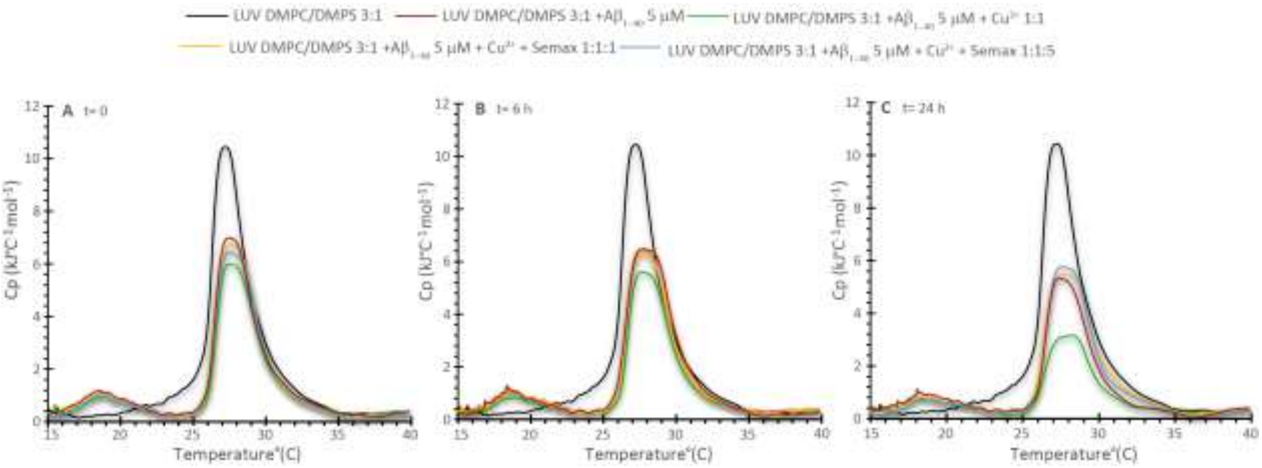

Figure S2. **Differential scanning calorimetry of LUV DMPC:DMPS 3:1.** Thermograms of LUV DMPC:DMPS 3:1 100 µM alone (black curve) and in presence of Aβ_{1-40} 5µM (red curve), Aβ_{1-40} 5µM + Cu^{2+} 5µM (green curve), Aβ_{1-40} 5µM + Cu^{2+} 5µM + Semax 5µM (yellow curve) and Aβ_{1-40} 5µM + Cu^{2+} 5µM + Semax 25µM (blue curve) at: Panel A time 0, Panel B 6 hours and Panel C 24 hours. All the experiments were performed in 10 mM MOPS buffer, 100 mM NaCl, pH 7.4.

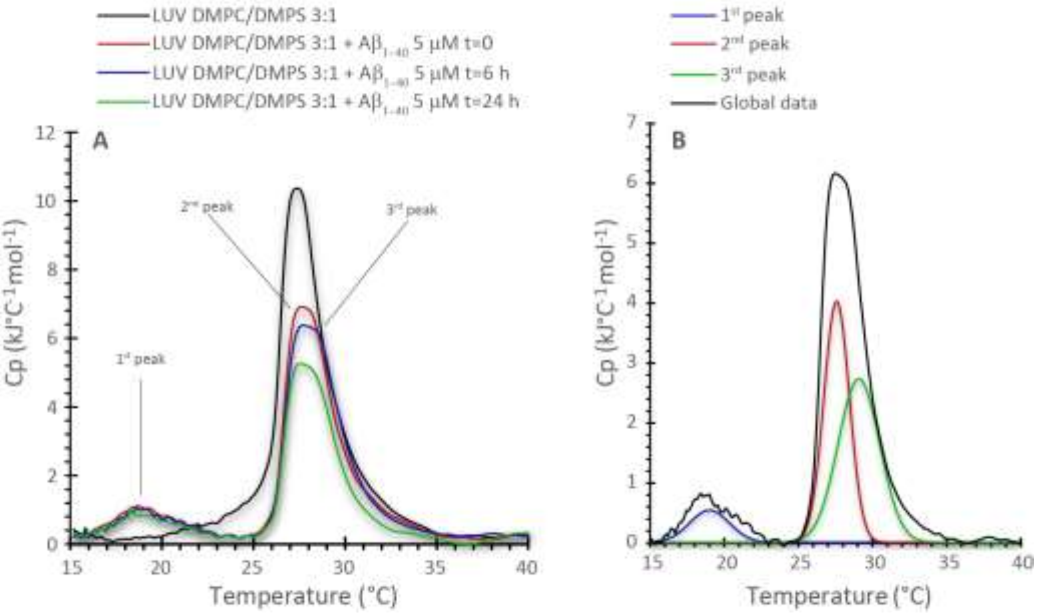

Figure S3. Panel A: thermograms of samples containing LUV DMPC:DMPS 3:1 100µM alone (black curve) and Aβ_{1-40} 5 µM at time 0 (red curve), after 6 hours (blue curve) and after 24 hours (green curve). The addition of Aβ_{1-40} induces phase segregation as evidenced by the appearance of three peaks. **Panel B:** each thermogram could be deconvoluted into the contribution of single peaks, the first one is correlated with domain in the membrane richer in zwitterionic lipids, the second one is correlated with domain with the average composition 3:1 and the third one is correlated to domains richer in negatively charge lipids.

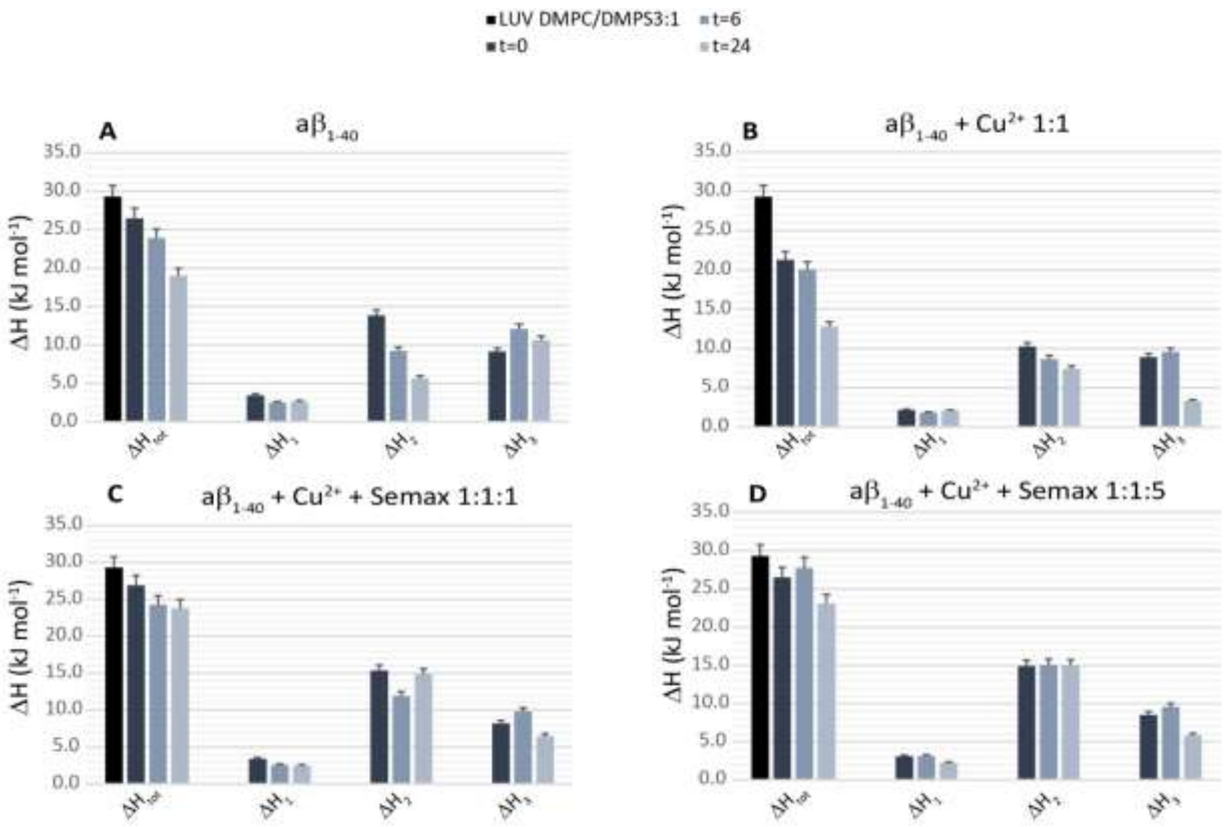

Figure S4. Comparison of enthalpy contribution. Contribution of ΔH of each peak over time for LUV DMPC:DMPS 3:1 in presence of Panel A: A$\beta_{1\text{-}40}$ 5μM, Panel B: A$\beta_{1\text{-}40}$ 5μM + Cu^{2+} 5μM, Panel C A$\beta_{1\text{-}40}$ 5μM + Cu^{2+} 5μM + Semax 5μM and Panel D: A$\beta_{1\text{-}40}$ 5μM + Cu^{2+} 5μM + Semax 25μM.

Sample	ΔH global (kJ mol^{-1})	T_1 (°C)	ΔH_1 (kJ mol^{-1})	T_2 (°C)	ΔH_2 (kJ mol^{-1})	T_3 (°C)	ΔH_3 (kJ mol^{-1})
DMPC/ DMPS 3:1	29.3 ± 1.5	-	-	27.5 ± 0.1	-	-	-
DMPC/ DMPS 3:1 + A$\beta_{1\text{-}40}$	26.5 ± 1.3	18.8 ± 0.1	3.5 ± 0.2	27.6 ± 0.1	13.8 ± 0.7	29.5 ± 0.1	9.2 ± 0.5
DMPC/ DMPS 3:1 + A$\beta_{1\text{-}40}$: Cu^{2+} 1:1	21.3 ± 1.1	19.0 ± 0.1	2.1 ± 0.1	27.6 ± 0.1	10.2 ± 0.5	29.2 ± 0.1	8.9 ± 0.4
DMPC/ DMPS 3:1 + A$\beta_{1\text{-}40}$: Semax: Cu^{2+} 1:1:1	26.9 ± 1.3	18.9 ± 0.1	3.4 ± 0.2	27.6 ± 0.1	15.3 ± 0.8	29.6 ± 0.1	8.2 ± 0.4
DMPC/ DMPS 3:1 + A$\beta_{1\text{-}40}$: Semax: Cu^{2+} 1:1:5	26.5 ± 1.3	18.8 ± 0.1	3.1 ± 0.2	27.7 ± 0.1	14.9 ± 0.7	29.6 ± 0.1	8.5 ± 0.4

Table S1. Thermodynamic parameter at t=0.

Sample	ΔH global (kJ mol^{-1})	T_1 (°C)	ΔH_1 (kJ mol^{-1})	T_2 (°C)	ΔH_2 (kJ mol^{-1})	T_3 (°C)	ΔH_3 (kJ mol^{-1})
DMPC/ DMPS 3:1	29.3 ± 1.5	-	-	27.5 ± 0.1	-	-	-
DMPC/ DMPS 3:1 + A$\beta_{1\text{-}40}$	23.9 ± 1.2	19.0 ± 0.1	2.1 ± 0.1	27.4 ± 0.1	9.2 ± 0.5	28.9 ± 0.1	12.1 ± 0.6
DMPC/ DMPS 3:1 + A$\beta_{1\text{-}40}$: Cu^{2+} 1:1	20.0 ± 1.0	19.0 ± 0.1	1.8 ± 0.1	27.6 ± 0.1	8.6 ± 0.4	29.1 ± 0.1	9.6 ± 0.5
DMPC/ DMPS 3:1 + A$\beta_{1\text{-}40}$: Semax: Cu^{2+} 1:1:1	24.3 ± 1.2	19.0 ± 0.1	2.6 ± 0.1	27.7 ± 0.1	11.9 ± 0.6	29.3 ± 0.1	9.8 ± 0.5
DMPC/ DMPS 3:1 + A$\beta_{1\text{-}40}$: Semax: Cu^{2+} 1:1:5	27.8 ± 1.4	18.9 ± 0.1	3.1 ± 0.2	27.8 ± 0.1	15.0 ± 0.8	29.1 ± 0.1	9.6 ± 0.5

Table S2. Thermodynamic parameter at t=6 h.

Sample	ΔH global (kJ mol^{-1})	T_1 (°C)	ΔH_1 (kJ mol^{-1})	T_2 (°C)	ΔH_2 (kJ mol^{-1})	T_3 (°C)	ΔH_3 (kJ mol^{-1})
DMPC/ DMPS 3:1	29.3 ± 1.5	-	-	27.5 ± 0.1	-	-	-
DMPC/ DMPS 3:1 + A$\beta_{1\text{-}40}$	19.0 ± 0.9	18.9 ± 0.1	2.6 ± 0.1	27.2 ± 0.1	5.7 ± 0.3	28.5 ± 0.1	10.6 ± 0.5
DMPC/ DMPS 3:1 + A$\beta_{1\text{-}40}$: Cu^{2+} 1:1	12.7 ± 0.6	18.8 ± 0.1	2.1 ± 0.1	27.8 ± 0.1	7.4 ± 0.4	29.6 ± 0.1	3.3 ± 0.1
DMPC/ DMPS 3:1 + A$\beta_{1\text{-}40}$: Semax: Cu^{2+} 1:1:1	23.8 ± 1.2	19.1 ± 0.1	2.5 ± 0.1	27.8 ± 0.1	14.8 ± 0.7	30.2 ± 0.1	6.4 ± 0.2
DMPC/ DMPS 3:1 + A$\beta_{1\text{-}40}$: Semax: Cu^{2+} 1:1:5	23.1 ± 1.1	19.3 ± 0.1	2.2 ± 0.1	27.8 ± 0.1	15.0 ± 0.7	30.1 ± 0.1	5.8 ± 0.2

Table S3. Thermodynamic parameter at t=24 h.

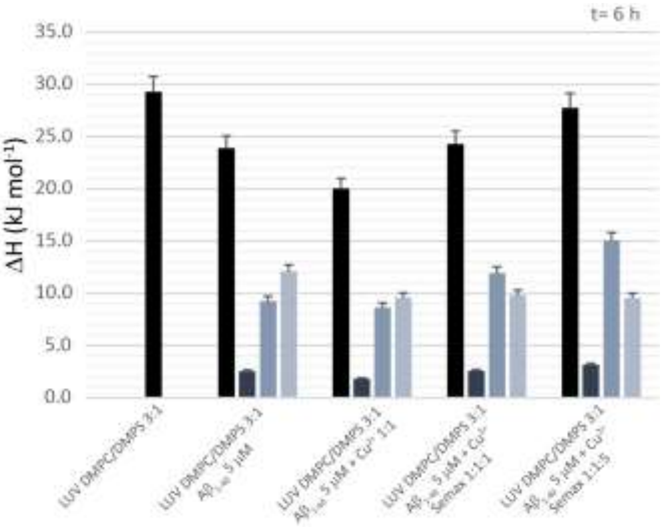

Figure S5. Comparison of enthalpy contribution. Contribution of ΔH of each peak for LUV DMPC:DMPS in presence of of $A\beta_{1-40}$ 5 µM, $A\beta_{1-40}$ 5 µM + Cu^{2+} 5 µM, $A\beta_{1-40}$ 5 µM + Cu^{2+} 5µM + Semax 5 µM and $A\beta_{1-40}$ 5 µM + Cu^{2+} 5 µM + Semax 25 µM at time 6h.

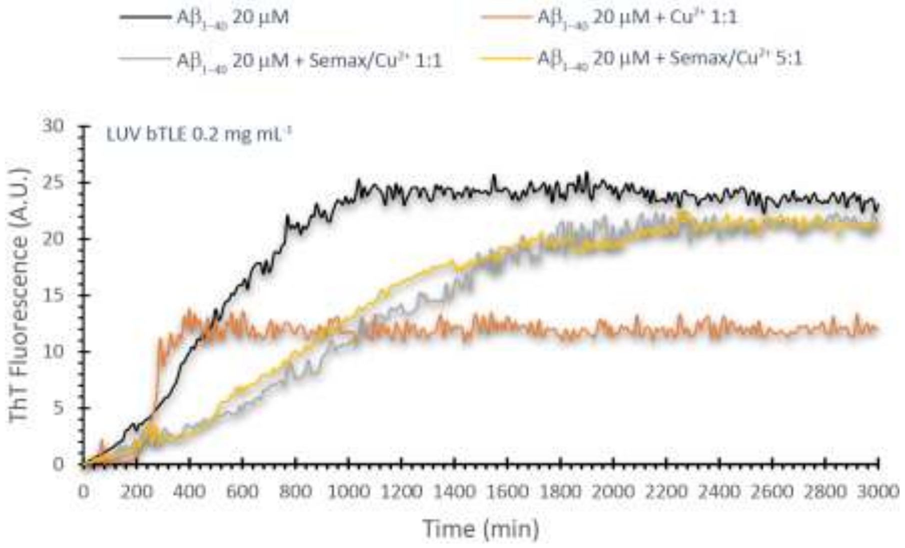

Figure S6. Thioflavin T assay in presence of bTLE LUVs. ThT traces of samples containing LUV bTLE 0.2 mg mL^{-1} and $A\beta_{1-40}$ 20 µM alone (black curve) and in presence of Cu^{2+} 20 µM (orange curve), preformed Semax : Cu^{2+} complex 1:1 (grey curve) and preformed Semax : Cu^{2+} complex 5:1 (yellow curve). All the experiments were performed at 37 °C in aCSF, pH 7.4. All traces are the average of three independent experiments.
